# Frequency of infection with *Mycoplasma suis* in gestating sows using qPCR on ten commercial French herds, and impact of the infection on clinical, haematological and biochemical parameters

**DOI:** 10.1186/s40813-020-00152-4

**Published:** 2020-07-01

**Authors:** Mathieu Brissonnier, Valérie Normand, Arnaud Lebret, Pierre-Yves Moalic, Anne-Sophie Guyomard, Véronique Bachy, Pauline Berton, Vincent Auvigne, Franck Bouchet, Gwenaël Boulbria

**Affiliations:** 1 Porc.Spective Swine Vet Practice - ZA de Gohélève, rue Joseph et Etienne Montgolfier, 56920 Noyal Pontivy, France; 2 Labofarm Finalab Veterinary Laboratory Group, 4 rue Théodore Botrel, 22600 Loudéac, France; 3 Orbio Finalab Veterinary Group, 12 rue du 35ème regiment d’aviation, 69500 Bron, France; 4 Ekipaj, 22 rue d’Assas, 49000 Angers, France

## Background

*Mycoplasma suis* (formerly known as *Eperythrozoon suis*) is a bacterium that causes infectious anaemia in pigs [9]. The syndrome has two main clinical forms: an acute form with high fever and anaemia, and a chronic form with multiple, non-specific clinical signs that can be more or less severe depending on strain virulence and pig susceptibility [16]. Since it first observation in the United States of America in 1932 [2, 10], the bacteria have been detected in pigs in all production areas in the world [3, 4, 11, 14, 15, 20]. The consequences of *M. suis* infection can be very significant, in particular for the breeding herd in the period around farrowing [1, 9, 16–18]. Infection with *M. suis* is also reported to result in decreased birth weights [23] and poor growth in post-weaning piglets [9]. Nevertheless, *M. suis* is not considered a major pathogen of swine. One reason for this is that the chronic form of the disease predominates and results in non-specific signs, which reduces the chances of making an aetiological diagnosis [15]. Secondly, *M. suis* is uncultivable, which makes it difficult to detect by conventional bacteriological techniques. For a long time, direct reading of a stained blood smear was the only available diagnostic method. This diagnostic limitation was a constraint for estimating its prevalence at regional and national level [5, 9, 15, 19]. The introduction of quantitative polymerase chain reaction (qPCR) tests has made it much easier to detect the bacterium. In a context of reduced antibiotic use and the availability of PCR, it has now become easier to study the impact of the bacteria on the health and performance of the sow herd. The first objective of our study was to assess the frequency of infection with *M. suis* infection in ten French herds. The second objective was to evaluate its impact on the production performances of sows and on the haematological and biochemical parameters in the farrowing period.

## Materials and methods

### Study design

The study was observational and cross-sectional. It was conducted during the winter of 2017/2018 in ten farms from the same veterinary practice located in Brittany (France). Herds were not randomly selected. Farms were chosen to be epidemiologically unrelated (all production types, herd sizes, different genetic and food suppliers). Herds were also selected for the absence of clinical outbreaks in sows and in weaning piglets (no infectious episodes affecting the animals observed) and the absence of treatment of sows with tetracyclines in the past 6 months. Moreover, we selected herds on the willingness of the farmer to participate and score monitoring criteria. All farms – two farrow-to-wean and eight farrow-to-finish operations – had an indoor production system (80 to 1000 sows, median = 335, Table 1).

**Table 1:** Characteristics of farms included in this study

Herd	Operation	N. of sows	N. of batches
1	Farrow-to-finish	550	20
2	Farrow-to-wean	1000	20
3	Farrow-to-finish	350	4
4	Farrow-to-finish	450	10
5	Farrow-to-finish	175	4
6	Farrow-to-finish	250	7
7	Farrow-to-wean	900	20
8	Farrow-to-finish	320	7
9	Farrow-to-finish	80	4
10	Farrow-to-finish	280	4

In each farm, 20 sows from the same batch were sampled in the week before farrowing (except one herd where only 19 sows could be sampled). The sample was stratified on the basis of gestation rank: 4 to 6 pregnant gilts, 3 to 6 sows in second gestation, 3 to 6 sows in third or fourth gestation and 4 to 9 sows in fifth or higher gestation. Health parameters were recorded for 8 days following farrowing.

### Sampling and data collection

Blood was collected by venipuncture (jugular vein). Three samples were collected in Vacutest® tubes (two EDTA tubes and one sodium fluoride and potassium oxalate tube) and submitted to the laboratory within 24 h under positive-cold conditions. The samples were taken at least 3 h after the last meal to avoid variations in blood sugar levels during the postprandial period [12, 13].

Clinical observations were made by the farmer. Numbers of total born and stillborn piglets were recorded. Rectal temperatures were measured once a day for 3 days after farrowing; sows were considered hyperthermic if the body temperature exceeded 39.5 °C at least once. Any signs of vaginal discharge in sows or diarrhoea in piglets were recorded once a day during the first week of lactation.

#### Detection of *Mycoplasma suis* by quantitative real-time PCR (qRT-PCR)

Deoxyribonucleic acid (DNA) was extracted from 200 μL EDTA blood samples using MagAttract 96 Cador Pathogen kit (Qiagen, Venlo, The Netherlands) following manufacturer’s instructions. Finally, DNA recovery was obtained in 100 μL elution buffer AVE and stored at − 20 °C.

A specific plasmid containing the targeted DNA sequence of *M. suis* was ordered (Eurofins, Luxembourg, Luxembourg): it contained the *M. suis* PCR target sequence. Dilutions were then used for absolute quantification assays. *M. suis* detection was achieved using a qRT-PCR test [6]. The primer, targeting 16S rDNA, was slightly modified (reverse primer 5′-CGCGAACACTTGTTAAGCAA-3′) so that the test could be run with Labofarm’s routine qRT-PCR thermal cycle, using Ultra-fast qPCR kit (Agilent Technologies, Les Ulis, France). Following sequence alignment of French field strains of M. suis, reverse primer has been shifted from a base to the 3′ end. The analytical specificity has been evaluated in silico against most of the septicemic agents in pigs (including *Streptococcus suis* and *Haemophilus parasuis*). Moreover, in silico specificity was evaluated for *Mycoplasma hyopneumoniae*, *Mycoplasma hyorhinis*, *Mycoplasma hyosynoviae*, *Mycoplasma flocculare*, *Actinobacillus pleuropneumoniae*, *Brachyspira intermedia*, *Brachyspira pilosicoli*, *Brachyspira innocens* and *Brachyspira hyodysenteriae*. Analytical specificity was also evaluated for *Streptococcus suis* and *Haemophilus parasuis* serotypes. No cross reaction was detected.

The quantification limit was achieved using *M. suis*-negative EDTA blood samples spiked with *M. suis* plasmid. The qRT-PCR is able to detect 10^6^ copies of 16S ribosomal DNA gene per mL of blood corresponding to 5 to 2.5 × 10^5^ bacteria per mL of blood. In this study, the results of qRT-PCR were analysed as a qualitative variable only.

#### Haematology

A complete blood cell count (CBC) was performed by impedance technique on Horiba ABX analyser (Horiba, Montpellier, France) on an EDTA-tube sample. The cellular components studied were red blood cells (RBC), white blood cells (WBC), and platelets. The RBC parameters measured were erythrocyte count, haematocrit, haemoglobin concentration, mean cell volume (MCV), mean corpuscular haemoglobin (MCH) and mean corpuscular haemoglobin concentration (MCHC). The analyser was calibrated daily using control blood (ABX Minotrol ND, Horiba).

#### Blood glucose test

The blood glucose test was performed by colorimetric technique on the Horiba Pentra 400 analyzer (Horiba, Montpellier, France); the samples in sodium fluoride and potassium oxalate tubes were used for this purpose.

### Statistical analysis

R software 3.5.1. (R Development Core Team 2019) was used. First, a multivariate analysis of the data using linear mixed-effect models was carried out. For all models, the statistical unit was the sow and the herd was set as a random effect. Interactions, heteroscedasticity and linearity of relations were checked. For the frequency of infection in selected herds, the dependent variable (outcome) was sow *M. suis* status and the explanatory variable was the gestation rank. For the study of haematological and health parameters, series of statistical models were built considering haematological and health parameters as dependent variables (outcome). The explanatory variables (predictor) were sow *M. suis* status, parity and number of total born piglets. For quantitative dependent variables (haematological parameters and stillborn piglets), linear models were used (glmmPQL procedure, family = “gaussian”). For binomial dependent variables (health parameters) logistic regression was used (glm procedure, family = “binomial”).

In a second step, parameters with a tendency (*p* < 0.10) for a statistical relationship with the sow *M. suis* status were selected. For these parameters, considering the importance of gilts in the immune balance of herds, the relationships between these parameters and the *M. suis* status were tested in the subpopulation of gilts. Wilcoxon and Fisher tests were used.

## Results

Samples were collected from 199 sows in ten farms. The analyses were carried out for 198 sows. Indeed for one sample, complete blood cells count could not be performed because of coagulation.

### Frequency of infection

Specific genetic material of *M. suis* was detected in 105 of the 198 samples (53%). No farm was free from *M. suis*. The observed intra-farm frequency of infection with *M. suis* ranged from 5 to 95% (median = 52%) (Table 2). Sows of gestation rank ≥5 were significantly less infected than pregnant gilts (Table 3).

**Table 2:** Intra-herd frequency of infection with *M.suis* and number of positive sows per gestation rank

	Number PCR Positive / Number sampled				
Herd	Gilts	Rank 2	Rank 3–4	Rank ≥5	Overall
1	2/6	1/3	4/6	3/5	50%
2	5/5	5/5	6/6	3/4	95%
3	3/5	3/5	4/6	3/4	65%
4	2/5	4/5	4/5	1/5	55%
5	4/5	0/5	2/5	3/5	45%
6	4/4	2/6	1/6	1/4	40%
7	2/5	3/4	4/5	1/5	53%
8	3/5	1/5	1/5	1/5	30%
9	0/4	0/3	1/3	0/9	5%
10	5/5	5/5	5/5	3/5	90%
Total	30/49	24/46	32/52	19/51	53%

**Table 3:** *M. suis* frequency of infection according to gestation rank

		PCR Positive		
Gestation rank	Total	n	%	*p*-value
1	49	30	61%	Ref.
2	46	24	52%	0.24
3, 4	52	32	62%	0.99
≥5	51	19	37%	0.01
	198	105	53%	

### Health parameters

#### Clinical and production parameters

Mean total born and mean stillbirth rate per litter was 16.1 (min = 2, max = 27) and 7% (min = 0, max = 38%), respectively. Diarrhoea in piglets and vaginal discharge in sows were observed in eight herds. Rectal temperature of the sows was recorded in ten herds. Incidences of diarrhoea, vaginal discharge and hyperthermia were 10, 10 and 15% of the sows, respectively (Table 4).

**Table 4:** Observed values for clinical parameters

	N. observed		N. positives	
	Herds	Sows	Herds	Sows
Diarrhoea (piglets)	8	153	4	16 (10%)
Vaginal discharge	8	154	4	16 (10%)
Hyperthermia (sows)	10	145	10	22 (15%)

In the whole population, the sow *M. suis* status was found to be significantly related to vaginal discharge. At equal gestation rank and total born, the risk of vaginal discharge was lower in infected sows. There was no statistically significant relation between sow *M. suis* status and sow hyperthermia and piglet diarrhoea during the first week of lactation. Only a tendency (*p* < 0,10) was observed for stillborns (Table 5).

**Table 5:** Influence on clinical parameters of *M. suis* status, gestation rank and litter size. A model is built for each outcome. For binary predictors (*M. suis* status and gestation rank), a positive coefficient indicates that the risk increases if the predictor is true. For Total-Born (TB), a positive coefficient means that the risk increases for each additional piglet

		Predictors				
		PCR Pos.	Rank 2	Rank 3, 4	Rank ≥5	TB
Outcomes						
Diarrhoea	Coeff	−0.12	−2.48	−3.74	− 28.94	− 0.02
	*p*-value	0.88	***0.00***	***0.00***	1.00	0.74
Vaginal discharge	Coeff	−1.46	−0.28	− 0.86	1.08	0.04
	*p*-value	***0.01***	0.69	0.26	*0.08*	0.53
Stillbirth	Coeff	0.01	0.00	−0.01	0.04	0.00
	*p*-value	0.42	0.94	0.70	***0.02***	***0.00***
Hyperthermia	Coeff	−0.03	0.62	1.04	1.22	0.08
	*p*-value	0.94	0.39	0.12	*0.07*	*0.10*

In the gilt sub-population, there was no statistically significant relation between gilt *M. suis* status and piglet diarrhoea, vaginal discharge and hyperthermia (Table 6). Nevertheless, a significantly higher rate of stillborns was observed in *M. suis* positive gilts compared to *M. suis* negative gilts. (Table 6).

**Table 6:** Influence of *M. suis* status, in the gilt sub-population, on clinical parameters

	*M. suis* neg	*M. suis* pos	*p*	Test
Diarrhoea	30%	56%	0.49	Fisher
Vaginal discharge	15%	8%	0.24	Fisher
Stillbirth	2.8%	7.4%	**0.02**	Wilcoxon
Hyperthermia	12.%	7.4%	0.63	Fisher

#### Haematological and biochemical parameters

The results of three blood glucose tests were outliers and therefore excluded (Table 7). No statistically significant relationship between the sow *M. suis* status and haematological parameters was found (*p* > 0.05) (Table 8). However, there was a tendency (*p* < 0.10) for haematocrit and lymphocytes to be higher among infected animals, at equal gestation rank and equal number of total births. Gestation rank had a significant effect for all haematological parameters. The number of total-born had a significant effect on number of red blood cells, haematocrit and MCHC. Finally, blood glucose did not seem to be related to the sow *M. suis* status, but there was also an effect of parity (Table 8).

**Table 7:** Observed values for haematological and biochemical parameters (*n* = 198 sows, except for blood glucose *n* = 195)

	Minimum	Median	Maximum
RBC (10^12^/L)	3.49	5.77	7.75
Haemoglobin (g/dL)	7.7	12.1	15.4
Haematocrit (%)	11.6	39.6	54.8
MCV (fL)	60	68	77
MCH (pg)	17.4	20.9	24.7
MCHC (g/dL)	24.5	30.6	33.6
Platelets (10^9^/L)	42	221.5	441
WBC (10^9^/L)	3.1	11.9	22.3
Neutrophils (10^9^/L)	0.3	7	14.4
Eosinophils (10^9^/L)	0	0.9	2.6
Basophils (10^9^/L)	0	0	0
Lymphocytes (10^9^/L)	1.3	3.3	6.7
Monocytes (10^9^/L)	0.1	0.6	1.5
Blood glucose (g/L)	0.51	0.9	1.17

**Table 8:** Influence on blood parameters of the *M. suis* status, gestation rank and litter size. A model is built for each outcome. For binary predictors (*M. suis* status and gestation rank), a positive coefficient means that the risk increases when the predictor is true. For Total-Born (TB), a positive coefficient means that the risk increases for each additional piglet

		Predictors				
		*M. suis* +	Gest. 2	Gest. 3, 4	Gest. ≥5	TB
Outcomes						
Erythrocytes	Coeff	0.07	−0.28	−0.40	−0.71	−0.02
	*p*-value	0.45	***0.01***	***0.00***	***0.00***	***0.03***
Haemoglobin	Coeff	0.16	−0.27	−0.50	− 0.55	−0.04
	*p*-value	0.37	0.21	***0.02***	***0.01***	*0.06*
Haematocrit	Coeff	1.17	−1.18	−2.25	−2.34	−0.17
	*p*-value	*0.09*	0.18	***0.01***	***0.01***	***0.03***
MCV	Coeff	0.27	1.54	2.25	4.49	0.00
	*p*-value	0.55	***0.01***	***0.00***	***0.00***	0.97
MCH	Coeff	0.01	0.47	0.52	1.57	0.02
	*p*-value	0.93	***0.03***	***0.01***	***0.00***	0.19
MCHC	Coeff	0.00	0.07	−0.26	0.35	0.04
	*p*-value	0.99	0.70	0.11	***0.04***	***0.01***
Leucocytes	Coeff	−0.09	−1.68	−2.39	−2.78	−0.03
	*p*-value	0.82	***0.00***	***0.00***	***0.00***	0.49
Neutrophils	Coeff	−0.41	−0.83	−1.16	−1.39	−0.01
	*p*-value	0.14	***0.02***	***0.00***	***0.00***	0.68
Eosionphils	Coeff	−0.03	−0.13	−0.23	− 0.18	0.00
	*p*-value	0.66	0.12	***0.01***	***0.03***	0.92
Lymphocytes	Coeff	0.24	−0.59	−0.81	−1.12	−0.01
	*p*-value	*0.08*	***0.00***	***0.00***	***0.00***	0.37
Monocytes	Coeff	0.03	−0.14	−0.18	−0.21	0.00
	*p*-value	0.16	***0.00***	***0.00***	***0.00***	0.15
Platelets	Coeff	−6.09	−13.66	−32.69	−57.56	0.61
	*p*-value	0.59	0.35	***0.02***	***0.00***	0.63
Glucose	Coeff	−0.01	−0.06	−0.04	−0.06	0.00
	*p*-value	0.69	***0.02***	0.14	***0.02***	0.92

The parameters for which at least one trend is observed are therefore haematocrit, lymphocyte count. The relationship between these parameters and the *M. suis* status is studied in the gilts sub-population. In gilts, the relationship is significant only for the lymphocyte count. (Table 9).

**Table 9:** Influence of *M. suis* status, in the gilt sub-population, on selected blood parameters

	n	*M. suis* neg	*M. suis* pos	*p*	test
Haematocrit	48	3.6	4.3	0.52	Wilcoxon
Lymphocytes	48	40.5	42.0	0.04	Wilcoxon

## Discussion

This study reports the frequency of infection with *M. suis* in gestating sows in ten herds selected in a French veterinary practice and the relationships between sow *M. suis* status and some clinical, haematological and biochemical parameters around farrowing. Data about the prevalence of *M. suis* are rare and *M. suis* infections have most likely been underdiagnosed, probably because of unclarified clinical signs observed in the field. In this study, only ten herds selected from a unique veterinary practice were sampled so these results cannot be extrapolated to the French pig population. None of the herd was free from *M. suis*. The proportion of PCR-positive sows in this study ranged from 5 to 95% with an average of 53%. In a recent study using a PCR-based diagnosis, conducted in Germany, the authors found an intra-herd prevalence of around 30% (ranging from 1 to 10 sows out of 10 sampled) in 21 farms [17]. The intra-herd frequency of infection with *M. suis* in our study is therefore quite similar. This high frequency of infection was observed even though the samples were taken before farrowing in our study to respect the animals’ well-being, whereas the best time to detect *M. suis* infection is in the days immediately after farrowing [21]. Finally, we found that frequency of infection with *M. suis* was significantly lower in sows in fifth or higher gestation than in pregnant gilts (37% vs. 61%), suggesting that an immunity against *M. suis* slowly develops as animals age. This parity effect was not observed by Stadler et al. [17]. Anaemia has been reported as the cardinal clinical sign of *M*. *suis* infection in pigs but our study did not reveal the relationship described in the literature [9, 15, 22] between sow *M. suis* infection and RBC parameters. Although a statistical trend (*p* < 0.10) was found for haematocrit, the effect is counter-intuitive because haematocrit was found to be higher in positive animals while anaemia was expected. These results are consistent with those observed in Brazil [4] and in Germany [17]. Finally, we did not observe a relationship, described under experimental conditions [16], between *M. suis* infection and a decrease in sow blood glucose levels. High fever has been described after experimental inoculation [16] but has not been observed in the field [18]. In this study, we observed no relationship between *M. suis* infection and hyperthermia within 3 days after farrowing. We found an increase in the leukocyte count in sows infected with *M. suis*, both in the overall population and in the gilt sub-population. This suggests a measurable immune response in sows during chronic *M. suis* infection. This finding is not in agreement with Henderson’s observation of panleukopenia in chronically infected sows [7] or with those of Stadler et al. who observed an increase in the number of leukocytes in *M. suis*-positive piglets but not in positive sows. We observed a significant increase in stillbirth rate in gilts, but this observation was not confirmed in the overall model. In a recent study, this effect on stillbirth rate was also described [17]. However, authors in their work did not make the distinction between sows and gilts as we did. Infection of sows with *M. suis* does not seem to promote the development of neonatal diarrhea. Apart from descriptions of dysgalactic or agalactic phenomena [18], no other studies have previously described this relationship, which is consistent with our results. Poor reproductive performances were previously described [8]. Our study showed a statistical trend (*p* ≤ 0.1) for a relation between *M. suis* infection and vaginal discharge during lactation. However, while an increased risk of discharge was expected with infection, the opposite was observed. This was not confirmed in the gilt population. Our study failed to demonstrate a significant impact of *M. suis* infection on production parameters and occurrence of clinical signs after farrowing, except an increase in stillbirth rate in gilts. However, we observed tendencies on some parameters that could be investigated in a largest sow population and herds.

## Conclusion

This observational study confirmed the relevance of qRT-PCR diagnosis of *M. suis* infection. The bacteria was detected in all farms and in all parities, suggesting that *M. suis* infection is probably widespread and enzootic. Nevertheless, in our study, based on recorded clinical signs and production, haematological and biochemical parameters, we could not demonstrate a significant impact of *M. suis* infection on health status and production performances of sow herds, except an increase in stillbirth rate in gilts.

## Data Availability

All datasets used in this study are available from the corresponding author on reasonable request.

## Abbreviations

CBC: Complete Blood Count analysis
DNA: Deoxyribonucleic acid
EDTA: Ethylenediaminetetraacetic acid
IAP: Infectious Anaemia of Pigs
*M. suis*: Mycoplasma suis
MCH: Mean Corpuscular Haemoglobin
MCHC: Mean Corpuscular Haemoglobin Concentration
MCV: Mean Cell Volume
qPCR: Quantitative polymerase chain reaction
RBC: Red Blood cells
RT-qPCR: Real-time quantitative polymerase chain reaction
TB: Total Born
WBCs: White Blood Cells
